# Overcoming challenges to dissemination and implementation of research findings in under-resourced countries

**DOI:** 10.1186/s12978-018-0538-z

**Published:** 2018-06-22

**Authors:** Richard J. Derman, Frances J. Jaeger

**Affiliations:** Global Affairs, Thomas Jefferson University, Philadelphia, PA USA

**Keywords:** Dissemination, Implementation, Diffusion, Evidence-based, Under-resourced, Evidence-supported interventions

## Abstract

Louis Pasteur once commented on the happiness that a scientist finds when, besides making a discovery, study results find practical application. Where health status is poor and resources are limited, finding such applications is a necessity, not merely a joy.

Dissemination, or the distribution of new knowledge gained through research, is essential to the ethical conduct of research. Further, when research is designed to improve health, dissemination is critical to the development of evidence-based medicine and the adoption of evidence-supported interventions and improved practice patterns within specific settings. When dissemination is lacking, research may be considered a waste of resources and a useless pursuit unable to influence positive health outcomes.

Effective translation of the findings of health research into policy and the practice of medicine has been slow in many countries considered low or lower middle-income (as defined by the World Bank). This is because such countries often have health care systems that are under-resourced (e.g., lacking personnel or facilities) and thus insufficiently responsive to health needs of their populations. However, implementation research has produced many tools and strategies that can prompt more effective and timelier application of research findings to real world situations.

A conscientious researcher can find many suggestions for improving the integration of research evidence into practice. First and foremost, the truthful reporting of results is emphasized as essential because both studies with desirable findings as well those with less than ideal results can provide new and valuable knowledge. Consideration in advance of the audience likely to be interested in study findings can result in suitable packaging and targeted communication of results. Other strategies for avoiding the barriers that can negatively impact implementation of research evidence include the early involvement of stakeholders as research is being designed and discussion before initiation of proposed research with those who will be affected by it. It is also important to recognize the role of education and training for ensuring the skills and knowledge needed for not only the conduct of high quality research but also for the meaningful promotion of results and application of research findings to achieve intended purposes.

“To him who devotes his life to science, nothing can give more happiness than increasing the number of discoveries, but his cup of joy is full when the results of his studies immediately find practical applications.” Louis Pasteur

## Background on dissemination and implementation

One of the inherent responsibilities of the ethical conduct of research is dissemination or the targeted distribution of newly acquired knowledge gained through research. When research is designed to improve health, dissemination of findings can be related to the following four ethical principles:

- Responsible use of public dollars,
- Benefit to participants/their cohorts/the public,
- Contributions to evidence-based knowledge and practice that maximize benefit and avert harm,
- Amelioration of health disparities [1].

The development of evidence-based medicine is dependent on dissemination. Further, when researchers examine their findings in the light of other published data supporting similar conclusions, this can create a platform for making clinical and policy decisions designed to improve health outcomes. When reporting research results, it is important to clearly state if an intervention produced its expected outcome. Level of certainty associated with the conclusion should also be indicated utilizing established criteria.

Research designed to improve patient care and health status is often directed at assessment of efficacy or effectiveness. Randomized controlled trials are most useful for determining efficacy of an intervention and its ability to produce therapeutic impact while evaluation of effectiveness is reliant on pragmatic trials that suggest how a given intervention or treatment may or may not work in a real-world situation. In the context of such health-related research, dissemination is essential and necessarily intertwined with implementation, or “the use of strategies to adopt and integrate evidence-based health interventions and change practice patterns within specific settings [2].” 

When drafting public policy, important variables of risk versus benefit are typically addressed, and the analysis should be supported by evidence. However, findings are often modulated by the projected costs associated with both implementation and dissemination; this is especially true in under-resourced settings. Additionally, factors associated with individuals expected to implement recommended strategies, as well as organizational factors, can impede integration of findings into practice, even when findings are based upon strong evidence. Implementation of findings can also take much longer than desirable and use of findings can be compromised when research is conducted without consideration of “real world” situations.

A concrete example of effective dissemination of evidence-based knowledge to expand the use of practices and achieve desirable outcomes is the Baby-Friendly Hospital Initiative (BFHI) [3]. A draft systematic review was prepared by the Evidence-based Practice Center and released by the US Agency for Healthcare Research and Quality for peer review. This systematic review rated the evidence for the effectiveness of this strategy in terms of influencing desirable outcomes as moderately strong [4]. While there is little disagreement about the benefits associated with the indicated outcomes of increasing rates of breastfeeding initiation, exclusivity for the first 6 months of an infant’s life, and continued duration for at least a year, not all babies are born into breastfeeding-friendly, hospital environments. Thus, there is the need for more research to determine other cost-effective means to achieve behavioral change among mothers and caregivers and to confirm the necessary conditions to improve both infant and maternal breastfeeding outcomes. This need is the reason that Thomas Jefferson University and research colleagues of Jawaharlal Nehru Medical College (JNMC), Belagavi (Karnataka, India) are conducting an exploratory study to assess if the involvement of peer counselors and use of mobile health (mHealth) educational tools have the potential for achieving greater adherence to evidenced-based breastfeeding recommendations.

## Why are dissemination and implementation research as well as diffusion research important?

Green and colleagues describe the approach that has been used to generate evidence-based guidelines to improve patient care as linear and funnel-like. Such an approach results when scientists undertake successive research to amass a large body of evidence which is then vetted and disseminated to policy makers and practitioners [5]. The linear approach has been challenged as it can be time-consuming and less than ideal, especially for under-resourced countries where health disparities are significant and delay in finding solutions to serious health problems can adversely affect many lives. Balas and Boren made the assertion in 2000 that it takes an estimated 17 years to fully integrate the results of research into practice and that typically only 14% of original research is used to the benefit of patient care [6]. While the time lag may have narrowed somewhat in recent years, the period of time for the translation of research into practice is generally considered too long. The low percentage of research successfully integrated into practice and used to improve care is also concerning. Critics have also suggested that efficacy research, best embodied by new drug trials, is often conducted in a manner that is not reflective of “real world” situations. For example, new drug trials are usually conducted on patients with no co-morbidities, an uncommon situation in many clinical practices.

Fortunately, an emphasis has been placed in recent years on the development of researchers who can focus on these types of problems and carry out implementation research or “the scientific inquiry into questions concerning implementation—the act of carrying an intention into effect, which in health research can be policies, programmes, or individual practices (collectively called interventions) [7].” This type of research is generally concerned with the users of research findings, not merely with the production of knowledge. Implementation research is important because it can inform:

- Quality improvement strategies,
- People who are influenced to change their behavior to have a healthier life,
- Communities who wish to change their condition [7].

Inadequate communication and dissemination can be a barrier to timely and effective implementation of research findings. Therefore, dissemination research is another means for increasing the probability of moving research discoveries to sustained adoption. This type of research has been defined as “the systematic study of how the targeted distribution of information and intervention materials to a specific public health audience can be successfully executed so that increased spread of knowledge about the evidence-based public health interventions achieves greater use and impact of the intervention [8].” 

Another type of research that has been discussed in dissemination and implementation literature is diffusion research, which involves “the systematic study of the factors necessary for successful adoption by stakeholders and the targeted population of an evidence-based intervention which results in widespread use (e.g., at the state or national level) and specifically includes the uptake or the penetration of broad scale recommendations through dissemination and implementation efforts, marketing, laws and regulations, systems research and policies [8].” Such research can be useful for detailing a plan that, if implemented, could have impact on a macro level.

## Does strong evidence ensure widespread relevance and adoption?

There is no shortage of examples from published work, including that resulting from our research in India, that the answer to this question is no. Research sponsored by the National Institute of Child Health and Human Development’s Global Network for Women’s and Children’s Health Research was conducted during the early 2000s in the Belagavi District of Karnataka State, India in a research area surrounding our research partner, Jawaharlal Nehru Medical College (JNMC). The research documented that misoprostol tablets given sublingually to mothers delivering in the home (the primary delivery location in India at the time) was 80% effective in reducing severe postpartum hemorrhage, for a cost of pennies per dose [9]. Although evidence was strong, why is it that the drug was not universally employed in similar settings? Will positive findings from the recently completed study of room-temperature-stable carbetocin suffer a similar fate? Or are there advocates who will disseminate findings showing that this uterotonic is especially efficacious for reducing postpartum hemorrhage? Will product champions emerge and utilize critical resources to ensure availability of carbetocin and its successful use, when appropriate?

Publications continue to repeat the adage, “We know what kills pregnant women and babies, and we know what will prevent most of their deaths.” Yet, countries are handicapped in implementing programs that work and are sustainable. Why? It is critical to ask this question as a means of removing the barriers to full implementation of evidence-based practices and achieving programmatic sustainability.

Throughout most of the world, the three leading causes of maternal mortality remain postpartum hemorrhage, hypertensive disorders of pregnancy, and sepsis. The research team at JNMC, in collaboration with US and UK partners, have performed extensive research in the first two areas of pregnancy complications [9–14] and are about to embark on studies tied to the prevention and early diagnosis of puerperal sepsis.

In the developed world (generally considered countries in the World Bank’s higher income categories), evidence-based protocols and policies have been established for the appropriate use and availability of multiple uterotonic agents, for assuring adequate access to prenatal care and an appropriate schedule of visits to detect and address pregnancy complications, and for promoting a concern for sterile technique and the indicated use of antibiotics. Adherence to these protocols and policies contribute to the relatively low rates of adverse pregnancy outcomes in developed countries. But introduction of similar protocols and policies may be met with resistance among health care providers in an under-resourced country or have little impact due to other barriers. Distribution of information about the research providing the evidence won’t be enough if no effort has been made to accommodate differences in the practice settings and provider capabilities between research sites and the under-resourced country.

Health research has increased in developing countries in recent years, resulting in the generation of many interventions proven to have beneficial impact on health status. However, knowledge about how to achieve scale-up, widespread dissemination and sustainability has not kept pace. Clearly, there is a need for dissemination and implementation research to narrow the gap between research and practice.

## Is dissemination applicable to research with unexpected or negative findings?

Dissemination remains a responsibility of investigators despite unexpected or negative research findings because such research can generate new knowledge, stimulate further research, and advance the search for solutions to significant health problems. An example which illustrates the importance of global health research and the need to recognize how local practices and infrastructures can affect outcomes is the recently published article, “A population-based, multifaceted strategy to implement antenatal corticosteroid treatment versus standard care for the reduction of neonatal mortality due to preterm birth in low-income and middle-income countries: the ACT cluster-randomized trial.” The study was designed to increase corticosteroids use among women who were at increased risk for a preterm birth. Since there was no debate about the efficacy of employing corticosteroids to reduce neonatal mortality in the developed world, this Global Network trial primarily focused on implementation and effectiveness. Despite increased use of antenatal corticosteroids among women delivering low-birthweight infants in the intervention group, neonatal mortality did not decrease in this group. Further, study results showed a surprising increase in harm among users of standard corticosteroid dosing [15]. Appropriately, these findings were responsible for delaying widespread promotion of the drug and stimulating additional research to determine why use of antenatal corticosteroids are associated with improved preterm birth outcomes in developed countries and what additional components should be added to an intervention strategy based upon antenatal corticosteroid use to achieve comparable results in low-resource settings.

Research can also identify interventions which achieve a desired outcome but also cause unintended consequences or compete long-term with the desired outcomes--e.g., increasing rates of non-indicated cesarean sections and inappropriate, widespread use of antibiotics. These are two practices, among many others, which may work at cross-purposes with the very effect that is desired. To report only findings with favorable impact and ignore dissemination of findings of harm would be a violation of principles associated with the ethical conduct of research involving human subjects.

## What can be done to increase the implementation of research findings and sustain the changes that are implemented as a result of research evidence?

Successful programmatic initiatives often begin at the top level, where executive leadership control available dollars, and then move downwards. Nevertheless, the importance of widespread education, relevant training and community mobilization cannot be overemphasized, and implementation research can be beneficial by focusing on the desired end-point and engaging those in the community whose support is required.

Our research initiatives in India have shown the importance of empowering community health workers as first-line team players in both the education of soon-to-be as well as presently pregnant women. Further, involvement of these village-based health workers in the early identification of women at risk for health complications has proven beneficial. Nevertheless, adequate dissemination of the knowledge gained through our research is necessary to influence scale-up and widespread diffusion to areas in India beyond our research area.

A growing body of peer-reviewed literature offers advice for translating science into evidence-based healthcare behaviors and practices that promote and improve health and can be sustained. Those writing about implementation science agree that success requires recognition that implementation spans multiple stages. Fixsen and colleagues suggested six implementation stages: exploration/adoption, installation, initial implementation, full implementation, innovation, and sustainability, [16] while Saldana specifies only three stages: pre-implementation, implementation, sustainability [17]. Despite variation in the number and description of stages, relevant publications identify similar activities or steps, which are considered essential for integration of research evidence into practice. Table 1 below provides recommendations and examples of activities that Woolf et al. have offered to increase implementation success.

**Table 1:** Recommendations for ensuring translation of research into health improvements [19]

Determine the user audience(s) for the research, define a user-oriented research agenda and appreciate the environment in which the users operate;	
Utilize objective methods of data collection and analysis and present data with sufficient transparency to establish trustworthiness;	
Package findings in a format appropriate for the audience and utilizing principles from graphic design, communication science, marketing, and the psychology of information processing;	
Engage stakeholders that have interest in an outcome and give voice to those who will be most directly affected before the research begins;	
Use effective communication science during interactions with decision makers, stakeholders and the public and reach the target audience(s) with the right message through the appropriate media.	

Table 2 presents useful recommendations that were developed by Glasgow and Emmons to enhance the movement of research into practice.

**Table 2:** Strategies for increasing the translation of research into practice [20]

Anticipate and address likely barriers to dissemination;	
Appreciate and integrate multiple types of evidence;	
Adopt research designs, such as practical clinical and behavioral trials across settings, that address concerns of clinicians and policymakers;	
Conduct broader evaluations that include multiple outcomes, address generalizability, and report on contextual factors;	
Do not expect a program to work perfectly initially, but plan for adaptation and refinement to fit local conditions and emerging issues that affect what constitutes effective interventions.	

Strategies for integration of research into practice can be complex; thus, it is beneficial to engage methodological support from the outset, to build necessary training requirements into stages of the implementation process, and to create research and service partnerships early. Neta et al. recommend an evaluation framework that highlights the domains required to enhance the value of implementation and dissemination research for end users [18]. Application of such an evaluation framework can play an important role in assuring that a program implemented for research on a small scale is appropriate for the larger population. While assuring sustainability of programs that integrate evidence-based health-promoting practices and behaviors is desirable, sustainability does not require a program to be frozen in time. Rather, a characteristic of sustainable programs is the ability to adapt and change when new knowledge emerges and needs change.

Finally, it is recommended that clinical trials addressing effectiveness (as a component of translational research) retain sufficient flexibility and incorporate intermediate process evaluations, reflective of the changing needs of the end users.
